# Giant metastasis to the choroid plexus of papillary thyroid cancer: case report and review of the literature

**DOI:** 10.1093/jscr/rjaf396

**Published:** 2025-06-09

**Authors:** Daniel Alejandro Vega-Moreno, Mónica Serrano-Murillo, Julio César López-Valdés, Gervith Reyes-Soto

**Affiliations:** Postgraduate Division, Faculty of Medicine, UNAM, Cto. de los Posgrados S/N, C.U., Coyoacán, 04510, Mexico City, Mexico; Neuro-Oncology Unit, Oncology Department, Instituto Nacional de Cancerología, Av. San Fernando No. 22, Col. Sección XVI Delegación Tlalpan, C.P. 14080, Mexico City, Mexico; Neuro-Oncology Unit, Oncology Department, Instituto Nacional de Cancerología, Av. San Fernando No. 22, Col. Sección XVI Delegación Tlalpan, C.P. 14080, Mexico City, Mexico; Neurosurgery Department, Hospital Central Sur de Alta Especialidad, PEMEX, Anillo Perif. 4091, Fuentes del Pedregal, Tlalpan, 14140, Mexico City, Mexico; Neuro-Oncology Unit, Oncology Department, Instituto Nacional de Cancerología, Av. San Fernando No. 22, Col. Sección XVI Delegación Tlalpan, C.P. 14080, Mexico City, Mexico

**Keywords:** choroid plexus metastases, central nervous system metastases, thyroid cancer metastases, intratumorally hemorrhage

## Abstract

Metastases to the choroid plexus are extremely rare, accounting for only 1% of brain metastasis cases. Among intraventricular tumors, the most frequent are papillary carcinomas, meningiomas, and papilloma, with rare metastatic lesions accounting for only 6% of all intraventricular lesions. We report the case of a patient with a giant metastatic lesion of papillary thyroid cancer with intratumorally hemorrhage in the choroid plexus of the left lateral ventricle, which was treated urgently with subtotal resection of the lesion and subsequent adjuvant treatment with radiotherapy. To date, we report the 10th case of primary choroid plexus metastasis of the thyroid.

## Introduction

Brain metastases are the most frequently reported intracranial tumors. However, metastases to the choroid plexus are especially rare, being difficult to diagnose, especially if there is no known diagnosis of primary cancer [1, 2]. There are very few cases of metastasis to the choroid plexus, and barely 1% of the cases are brain metastasis. The most recent review by Garrido *et al*. mentions only 94 cases reported to date, where the most frequent histological strain was renal in 45% of the cases found in the literature [3]. The form of presentation has been heterogeneous, from cases reported as multifocal lesions, even lesions with intraventricular or tumoral hemorrhage. No giant lesions such as ours have been reported below [4]. We report the case of a patient with a giant metastatic lesion of papillary thyroid cancer with intratumorally hemorrhage in the choroid plexus of the left lateral ventricle. We report the 10th case of these characteristics recorded in the literature.

## Case report

**Figure 1:**
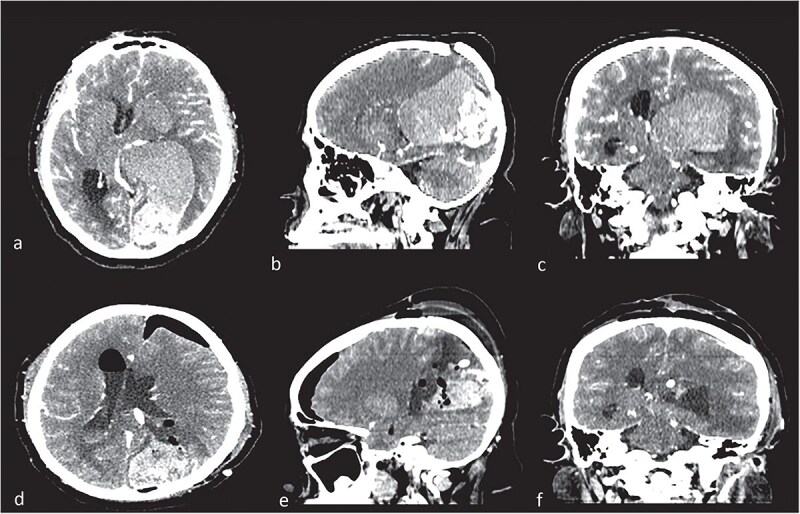
Contrast computed tomography (CT). (a–c) Intraventricular tumor, with a high volume, with the lateral ventricle shape. Different density in the tumor meaning intratumorally hemorrhage. (c–e) Control CT with resection of low-density lesion.

A 56-year-old female patient with a surgical history of drainage of a left parietal subdural hematoma 3 months prior to the current condition. The hematoma was spontaneous and was resolved by drainage without complications. In addition, the patient had a history of paraphasia, occasional headaches, and echolalia 1 month prior to her current condition. Fifteen days prior to her admission, a fine needle biopsy was performed on the thyroid due to a neck lesion with increased volume and pain. The pathology study reported papillary carcinoma of the thyroid. She was admitted as scheduled for thyroidectomy and wide neck resection. Upon admission, she presented sudden neurological deterioration, intense headache, motor aphasia, and weakness of the right side of the body. An urgent simple and contrast-enhanced cranial tomography was performed with the following findings (Fig. 1).

**Figure 2:**
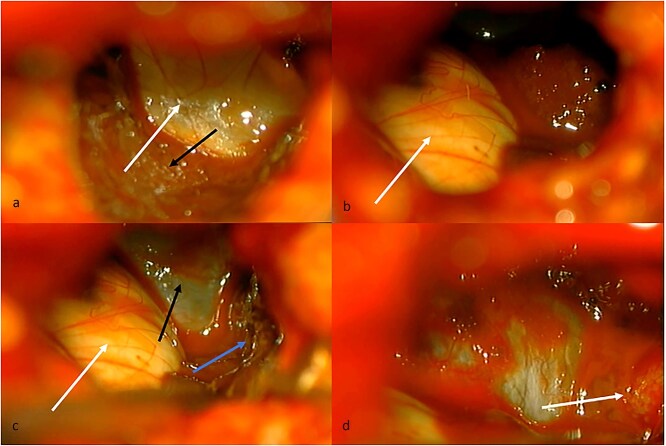
(a) Intraventricular view. White arrow, floor of the left lateral ventricle. Black arrow, tumor tissue covering thalamus. (b) White arrow, thalamus in the anterior, and medial limit of the lateral ventricle. (c) White arrow, thalamus. Black arrow, floor of the lateral ventricle. Blue arrow, tumor tissue covering lateral limit of lateral ventricle. (d) White arrow, choroid fissure with choroid plexus, and choroidal artery.

Given the presence of hemorrhage from a probable metastatic lesion on the left parietal, it was decided to undergo craniotomy and tumor resection. It was decided to perform an approach on the previous parietal craniotomy. The patient was placed in the concorde position, and the previous incision was extended. Likewise, the previous craniotomy was extended toward the occipital base. A transulcal posterior parietal approach was performed. The ventricular ependyma was found 5 mm from the parietal cortex, which was opened, and black, chocolate-colored liquid from the left lateral ventricle was found. A dark brown lesion was also observed, adhered to the walls of the lateral ventricle, toward the thalamus, floor of the ventricle, ventricular atrium, and multiple implant areas toward the choroid fissure, choroid plexus, and choroid artery. A subtotal resection was performed since the solid portion of the tumor was found with abundant blood vessels and adhered to the choroid plexus, with abundant bleeding (Fig. 2). The histopathological report confirmed a metastatic lesion in the brain of the thyroid papillary gland. The patient improved neurologically in the immediate postoperative period and was discharged 2 days later with improved consciousness and strength. She continued her oncological treatment, and treatment of the cranial metastatic lesion was complemented with radiotherapy (Fig. 1).

## Discussion

Brain metastases from thyroid carcinomas are extremely rare, reported in ~1% of all thyroid cancers [5]. The most frequent clinical presentations reported in choroid plexus metastases are hydrocephalus, intraventricular hemorrhage, impaired alertness, or hypertensive cranial syndrome. However, reports from advanced imaging studies, such as positron emission tomography-computed tomography (PET-CT), show that asymptomatic lesions are showing [6–8]. Within intraventricular tumors, the most frequent are papillary carcinomas, meningiomas, and papilloma, with metastatic lesions being infrequent, accounting for only 6% of all intraventricular lesions [9, 10].

Metastases to the choroid plexus have been reported as case reports or series with very little information. The mean age has been described as 62 years, with the lateral ventricles being mostly affected as opposed to the third ventricle [11]. Although it has been reported that surgery, especially total resection of the lesion, is the best prognostic indicator, other therapeutic options should be considered in the comprehensive management of these patients, such as chemotherapy to control the primary and even radiotherapy with whole brain radiotherapy (WBRT) and stereotactic radiosurgery (SRS) [12].

The treatment of metastatic lesions to the choroid plexus should be complemented with radiotherapy regardless of the primary histology. Radiosurgery has shown promising results in terms of survival, 25 + −23 months after surgery, in lesions from 0.9 to 4.1 cm in size [13].

Of the cases reported to date, three were managed only with biopsy, four underwent surgery achieving complete resection; in one case, a subtotal resection was achieved, and in another, surgery was rejected. Seven of the nine cases and 11 lesions reported were treated with adjuvant radiotherapy. Seven cases (77.7%) of the nine have papillary variants, while only two have been reported as follicular ones. We present the case of one variant (Table 1).

**Table 1:** Cases reported up to date of choroid plexus metastases of thyroid cancer

**Author**	**Year**	**Kind of thyroid cancer**	**Surgical brain treatment**	**Prognosis and survival**
Zhang *et al*. [12]	2008	Follicular	Interhemispheric transcallosal approach/subtotal resection	18 months at least/no adjuvanted with radiotherapy
Wasita *et al*. [5]	2010	Papillary (two lesions, one subthalamic, and the main in right ventricle)	Occipital transcortical approach/total resection	2 years/no radiotherapy. Was treated one more subthalamic lesion with SRS
Heery *et al*. [14]	2012	Papillary	Parieto-occipital craniotomy/resection	13 months with stereotactic radiotherapy
Palot Manzil *et al*. [15]	2014	Papillary	Any surgical treatment	Gamma knife/no data about survival
Healy *et al*. [16]	2014	Hürthle cell papillary	Gross total resection after 6 years of radiotherapy (no approach specified)	8 years at least/gamma knife in two times
Umehara *et al*. [10]	2015	Papillary	Endoscopic biopsy	8 months/SRT 33 Gy
Sharifi *et al*. [9]	2015	Papillary (two lesions in mirror)	Intraparietal posterior parasagittal approach/total resection	Radiotherapy to contralateral lesion. 6 months of follow-up
Kitagawa *et al*. [1]	2015	Follicular	Transcortical parietal posterior biopsy	14 months after radiotherapy 60 Gy
Beach *et al*. [4]	2021	Papillary	Endoscopic biopsy	3 weeks of survival/palliative care

## Conclusion

We are not sure about the history of the subdural hematoma since the patient was previously treated in another hospital unit. However, we theorize that the spontaneous subdural hematoma was secondary to tumor burden and that since then (3 months prior to her current condition), the patient had a metastatic lesion on the left parietal side that had debuted as a subdural hematoma. Although metastatic lesions to the choroid plexus are rare, thyroid metastases in this location are even rarer, with only a few case reports reported in the literature. This lesson is the first report of this size. A giant lesion with acute neurological deficit with multiple implants throughout the extent of the choroid plexus of the lateral ventricle. Although total resection was not achieved, given the characteristics of the lesion, we achieved adequate decompression and symptom control. We report the 10th case reported to date and the first of a giant size in this location.
